# Supplementary material for: Enhanced Tensile Properties of Multi-Walled Carbon Nanotubes Filled Polyamide 6 Composites Based on Interface Modification and Reactive Extrusion
Source: Polymers (Basel). 2020 Apr 25;12(5):997. doi: 10.3390/polym12050997 (PMC7284767; doi:10.3390/polym12050997)
Supplement: Supplementary file 1 [file polymers-12-00997-s001.pdf]

Supplementary Materials

Enhanced Tensile Properties of Multi-Walled Carbon Nanotubes Filled Polyamide 6 Composites Based on Interface Modification and Reactive Extrusion

Min Park [1,2], Ji-un Jang [1], Jong Hyuk Park [1], Jaesang Yu [1,*], Seong Yun Kim [3,*]

1 Korea Institute of Science and Technology (KIST), Seoul 02792, Korea; jju204@naver.com (J.J.); hyuk0326@kist.re.kr (J.H.P.)

2 KHU-KIST Department of Converging Science and Technology, Kyung Hee University, Seoul 02447, Korea; minpark@kist.re.kr

3 Division of Polymer-Nano & Textile Engineering, Chonbuk National University, Jeonju-si 54896, Korea

* Correspondence: jamesyu@kist.re.kr (J.Y.), sykim82@jbnu.ac.kr (S.Y.K.)

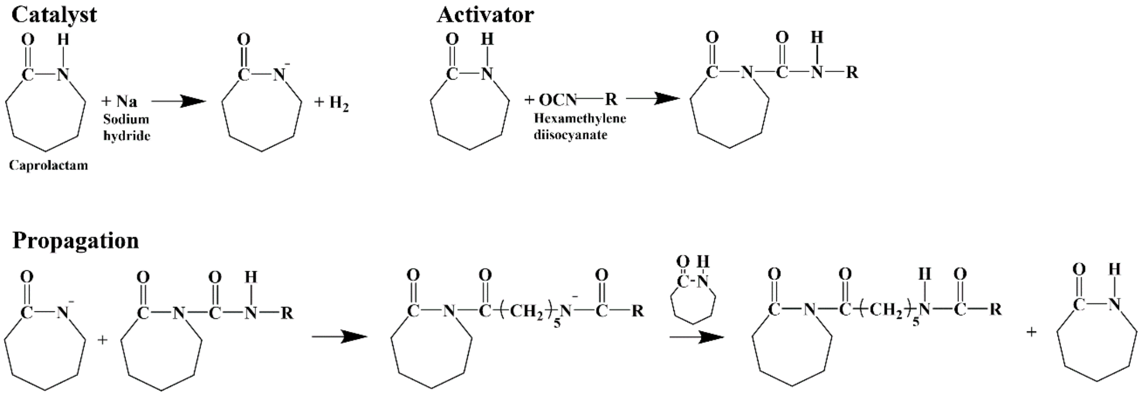

Figure S1. Anionic polymerization of ε-caprolactam.

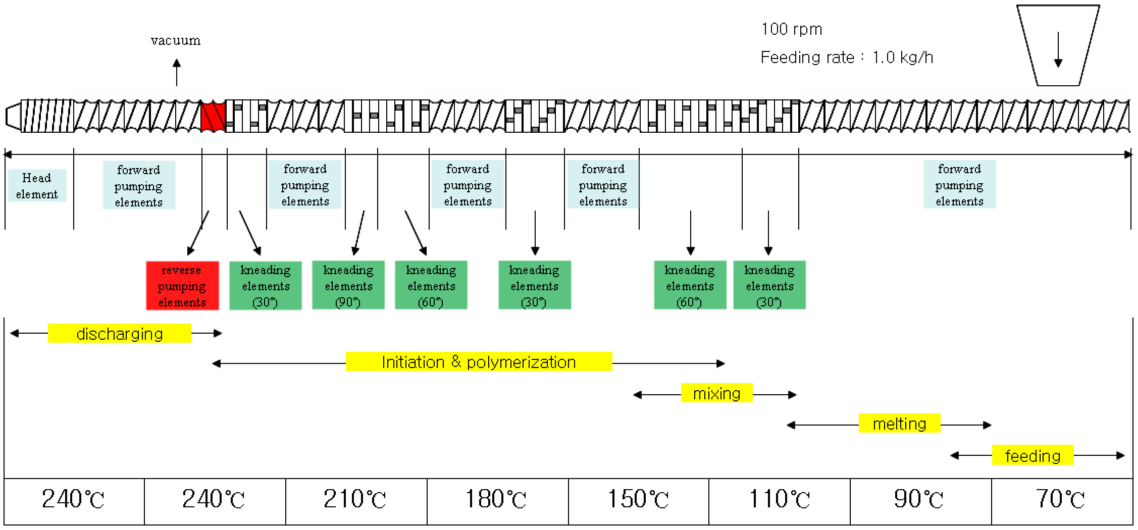

Figure S2. Screw configuration and barrel temperature.

The manufacturing conditions should be set in consideration of the mass transfer, heat transfer, reaction, and flow field interactions in the reactive extrusion process. In the reactive extrusion, the configuration, rpm, barrel temperature, and feeding rate of the screw affect the molecular weight and molecular weight distribution of the polymerized PA6 polymers. By performing preliminary experiments for the anionic polymerization under various process conditions, the optimized process conditions as well as screw configuration were set as shown in Figure S2, considering the processability and specimen conditions.

Reaction Kinetics Expression of Wittmer and Gerrens

$$r = [CL]_0 \frac{1}{T_f - T_0}\frac{dT}{dt} = Ae^{-E/RT}[C][A][CL]_0 \frac{T_f - T}{T_f - T_0} \quad \text{(S1)}$$

where $[A]$: activator concentration, $[C]$: catalyst concentration, $[CL]_0$: initial ε-caprolactam concentration, r: reaction rate, T: temperature at time t, T_0: initial temperature, T_f: final temperature, A: frequency factor, E: activation energy, and R: gas constant.

Degree of Conversion

$$\beta = \frac{Q}{\Delta H} \quad \text{(S2)}$$

where $Q = c_p(T - T_0)$ and $\Delta H = c_p(T_f - T_0)$, Q: heat of reaction, ΔH: enthalpy of reaction, and c_p: specific heat. The heat (Q) was obtained with the aid of the specific heat c_p and the temperature increase ($T - T_0$) above the initial temperature and the enthalpy of reaction (ΔH) was obtained from the maximum temperature increase $(T_f - T_0)$.

$$\beta = \frac{T - T_0}{T_f - T_0} \quad \text{(S3)}$$

Prediction of Tensile Properties

Young's modulus and tensile strength of the composite were calculated based on the Halpin–Tsai equation and the rule of mixture, respectively [1–3]. Young's modulus of the composite with randomly dispersed MWCNTs was calculated based on the Halpin–Tsai equation.

$$E_c = \left[\frac{3}{8}\frac{1 + 2(l_{MWCNT}/d_{MWCNT})\eta_L V_{MWCNT}}{1 - \eta_L V_{MWCNT}} + \frac{5}{8}\frac{1 + 2\eta_\perp V_{MWCNT}}{1 - 2\eta_\perp V_{MWCNT}}\right] E_m \quad \text{(S4)}$$

where $\eta_L = \frac{(E_{MWCNT}/E_m)-1}{(E_{MWCNT}/E_m)+2(l_{MWCNT}/d_{MWCNT})}$ and $\eta_\perp = \frac{(E_{MWCNT}/E_m)-1}{(E_{MWCNT}/E_m)+2}$, E_c: Young's modulus of the composite, E_m: Young's modulus of the matrix, E_m = 0.3574 GPa, E_{MWCNT}: Young's modulus of MWCNT, E_{MWCNT} = 470 GPa [4], l_{MWCNT}: length of MWCNT, l_{MWCNT} = 10 μm, and d_{MWCNT}: diameter of MWCNT, d_{MWCNT} = 10 nm, V_{MWCNT}: volume fraction of MWCNTs in the composite, ρ_{MWCNT}: density of MWCNT, ρ_{MWCNT} = 1750 Kg/m^3 [5], ρ_m: density of the polymer matrix, and ρ_m = 1140 Kg/m^3 [6]. Based on the values of E_{MWCNT} and E_m, the constant η_L and $\eta_\perp$ are η_L = 0.40; $\eta_\perp$ = 1.0

V_{MWCNT} of the composite can be calculated as follows:

$$V_{MWCNT} = (1.0\%/\rho_{MWCNT})/(1.0\%/\rho_{MWCNT} + 99.0\%/\rho_m) = (1.0\%/1750)/(1.0\%/1750 + 99.0\%/1140) = 0.65\%$$

Young's modulus of the composite incorporated with randomly dispersed MWCNT of 1 wt% content can be calculated with the following equation:

$$E_c = \left[\frac{3}{8}\frac{1 + 2 * (10000/10) * 0.40 * 0.65\%}{1 - 0.40 * 0.65\%} + \frac{5}{8}\frac{1 + 2 * 1.0 * 0.65\%}{1 - 2 * 1.0 * 0.65\%}\right] 0.35 = 1.058 \text{ GPa}$$

Tensile strength of the composite was calculated based on the rule of mixture.

$$\sigma_c = \sigma_{MWCNT} V_{MWCNT} + \sigma_m V_m \quad \text{(S5)}$$

where σ_c = tensile strength of the composite, σ_{MWCNT}: tensile strength of MWCNT, σ_{MWCNT} = 20 GPa [4], σ_m: tensile strength of the matrix, σ_m = 0.062 GPa, V_{MWCNT}: volume fraction of MWCNTs in the composite, V_m: volume fraction of the matrix in the composite. V_m of the composite can be calculated as follows:

$$V_m = (99.0\%/\rho_m)/(1.0\%/\rho_{MWCNT} + 99.0\%/\rho_m) = (99.0\%/1140)/(1.0\%/1750 + 99.0\%/1140) = 99.35\%$$

Tensile strength of the composite incorporated with MWCNT of 1 wt% content can be calculated with the following equation:

$$\sigma_c = \sigma_{MWCNT} V_{MWCNT} + \sigma_m V_m = 20 * 0.65\% + 0.062 * 99.35\% = 195 \text{ MPa}$$

References

[1] Halpin Affdl, J.C.; Kardos, J.L. The Halpin-Tsai equations: A review. *Polym. Eng. Sci.* **1976**, *16*, 344–352.

[2] O'Regan, D.F.; Akay, M.; Meenan, B. A comparison of Young's modulus predictions in fibre-reinforced-polyamide injection mouldings. *Compos. Sci. Technol.* **1999**, *59*, 419–427.

[3] Agarwal, B.D.; Broutman, L.J.; Chandrashekhara, K. Analysis and performance of fiber composites, Analysis and performance of fiber composites.; Wiley: NY, USA, 1980.

[4] Yu, M.-F.; Lourie, O.; Dyer, M.J.; Moloni, K.; Kelly, T.F.; Ruoff, R.S. Strength and breaking mechanism of multiwalled carbon nanotubes under tensile load. *Science* **2000**, *287*, 637–640.

[5] Shaffer, M.S.P.; Windle, A.H. Fabrication and characterization of carbon nanotube/poly(vinyl alcohol) composites. *Adv. Mater.* **1999**, *11*, 937–941.

[6] Bilotti, E.; Zhang, R.; Deng, H.; Quero, F.; Fischer, H.R.; Peijs, T. Sepiolite needle-like clay for PA6 nanocomposites: An alternative to layered silicates? *Compos. Sci. Technol.* **2009**, *69*, 2587–2595.
